# Maternal alcohol use, adverse neonatal outcomes and pregnancy complications in British Columbia, Canada: a population-based study

**DOI:** 10.1186/s12884-021-03545-7

**Published:** 2021-01-22

**Authors:** Svetlana Popova, Danijela Dozet, Graham O’Hanlon, Valerie Temple, Jürgen Rehm

**Affiliations:** 1 Institute for Mental Health Policy Research, Centre for Addiction and Mental Health, 33 Ursula Franklin Street, Toronto, ON M5S 2S1 Canada; 2 Dalla Lana School of Public Health, University of Toronto, 155 College Street, Toronto, ON M5T 3M7 Canada; 3 Factor-Inwentash Faculty of Social Work, University of Toronto, 246 Bloor Street W, Toronto, ON M5S 1V4 Canada; 4 Institute of Medical Science, Faculty of Medicine, University of Toronto, Medical Sciences Building, 1 King’s College Circle, Toronto, ON M5S 1A8 Canada; 5 Surrey Place, 2 Surrey Place, Toronto, ON M5S 2C2 Canada; 6 Institute of Clinical Psychology and Psychotherapy & Center of Clinical Epidemiology and Longitudinal Studies, Technische Universität Dresden, Chemnitzer Str. 46, 01187 Dresden, Germany; 7 Department of Psychiatry, University of Toronto, 250 College Street, Toronto, ON M5T 1R8 Canada

**Keywords:** Alcohol, Epidemiology, Perinatal, Pregnancy, Medical disorders in pregnancy; substance misuse in pregnancy, Birth outcomes

## Abstract

**Background:**

The current study aimed to estimate the prevalence of alcohol use identified as a risk factor during pregnancies by the antenatal care providers, resulting in live births in British Columbia (BC) and to examine associations between alcohol use, adverse neonatal outcomes, and pregnancy complications.

**Methods:**

This population-based cross-sectional study utilized linked obstetrical and neonatal records within the BC Perinatal Data Registry (BCPDR), for deliveries that were discharged between January 1, 2015 and March 31, 2018. The main outcome measures were alcohol use identified as a risk factor during pregnancy, associated maternal characteristics, pregnancy complications, and adverse neonatal outcomes. Estimates for the period and fiscal year prevalence were calculated. Chi-square tests were used to compare adverse neonatal outcomes and pregnancy complications by alcohol use during pregnancy identified as a risk factor. Logistic regression was used to examine the association between alcohol use identified as a risk factor during pregnancy and adverse neonatal outcomes and pregnancy complications, after adjusting for identified risk factors.

**Results:**

A total of 144,779 linked records within the BCPDR were examined. The period prevalence of alcohol use during pregnancy identified as a risk factor was estimated to be 1.1% and yearly prevalence was 1.1, 1.1, 1.3 and 0.9% from the 2014/2015 to 2017/2018 fiscal years, respectively.

Alcohol use identified as a risk factor was associated with younger maternal age, fewer antenatal visits, being primiparous, a history of mental illness, substance use and smoking.

Neonates with alcohol use during pregnancy identified as a risk factor had greater odds of being diagnosed with: “low birth weight (1000-2499g)” (ICD-10: P07.1; aOR = 1.25; 95% CI: 1.01, 1.53), “other respiration distress of newborn” (ICD-10: P22.8; aOR = 2.57; 95% CI: 1.52, 4.07), “neonatal difficulty in breastfeeding” (ICD-10: P92.5; aOR = 1.97; 95% CI: 1.27, 2.92) and “feeding problems, unspecified” (ICD-10: P92.9; aOR = 2.06; 95% CI: 1.31, 3.09).

**Conclusions:**

The prevalence of alcohol use during pregnancy identified as a risk factor was comparable to previous estimates within the BCPDR. Identified prenatal alcohol exposure was associated with notable differences in maternal and neonatal characteristics and adverse neonatal outcomes. More consistent, thorough screening and prevention efforts targeting alcohol use in pregnancy are urgently needed in Canada.

**Supplementary Information:**

The online version contains supplementary material available at 10.1186/s12884-021-03545-7.

## Background

Maternal consumption of alcohol during pregnancy is a well-known risk factor for adverse neonatal outcomes [1–4]. Estimated rates of alcohol consumption by women during pregnancy can vary depending on factors such as the population studied and data collection methods; however, overall global prevalence is estimated to be about 10% [5]. In recent years, the prevalence of alcohol use and binge drinking among women of childbearing age have been increasing in the majority of countries globally, including Canada [6].

Prenatal alcohol exposure (PAE) can affect multiple aspects of infant health and development [7–9]. Possible adverse neonatal outcomes associated with maternal alcohol consumption include congenital and physical abnormalities such as cardiovascular, musculoskeletal, and craniofacial defects [4, 9]; medical conditions such as respiratory distress and convulsions [10, 11]; and early care challenges such as sleeping and feeding problems [1]. PAE may also have an adverse effect on fetal growth for parameters such as weight, length, and head circumference; however, the research evidence for growth deficits is mixed, especially at low to moderate levels of exposure [12].

PAE does not appear to affect all pregnancies equally, suggesting that a number of additional factors may also be involved in neonatal outcomes. These may include the amount of alcohol consumed by the mother, nutrition, genetic factors, other prenatal exposures (e.g., tobacco, cannabis), pregnancy complications, and maternal attributes such as age, parity or existing health conditions [12, 13].

Data from the 2006 Maternity Experiences Survey suggested that in British Columbia (BC), 38% of pregnant women received prenatal care from obstetricians and gynaecologists, 52% received care from family physicians and general practitioners; and 10% from midwives and nurses [14]. Though health care services are free in Canada, prenatal care from obstetricians may be less accessible to women in rural and remote communities in BC, who often solely receive prenatal care from family physicians [15]. Canadian guidelines (2020) for screening and counselling for alcohol and pregnancy [16] suggest that all women should be screened for alcohol use. However, currently, differences exist between provincial/territorial prenatal care providers in implementing alcohol use screening [17–19], which makes the data on the prevalence of PAE incomparable across Canada.

Prenatal services in British Columbia (BC), Canada are administered at a population level by Perinatal Services BC (PSBC), a provincial agency mandated with the coordination of prenatal and newborn screening programs. PSBC collects and analyzes maternal and neonatal information using the British Columbia Perinatal Data Registry (BCPDR), a quality-controlled database containing clinical information on nearly 100% of births throughout the province of BC from all service providers and obstetric facilities. The BCPDR collects standardized data on antenatal, intrapartum, postpartum and neonatal information, including ICD-10 codes indicated at birth and neonatal encounters.

Using population-based data from the BCPDR [17], this study sought to estimate the prevalence of pregnancies in which maternal alcohol use was identified as a risk factor and examine possible associations between alcohol use in pregnancy, adverse neonatal outcomes, and pregnancy complications. In addition, the study examined maternal attributes and risk factors for alcohol use identified as a risk factor during pregnancy.

## Methods

### Data source

Maternal and neonatal records were obtained from PSBC for discharge dates between January 1, 2015 and March 31, 2018 [17]. Exclusion criteria for neonatal records included therapeutic abortions, stillbirths, unlinked maternal/infant records and records where the sex of the newborn was listed as unidentified.

### Prenatal exposures, covariates and outcomes

Maternal alcohol use and binge drinking (four or more drinks per occasion) during pregnancy were recorded during antenatal visits when alcohol use was identified as a risk factor by the antenatal care provider. Alcohol use was initially identified through maternal self-report in most cases, but women only reported on alcohol use following pregnancy recognition. Information on binge drinking was available only among women with alcohol use identified as a risk factor during the respective pregnancy.

Other substance use was recorded during antenatal visits by the antenatal care provider only when it was identified as a risk, during the respective pregnancy. Data on substance use included heroin, methadone, cocaine, marijuana, prescription drugs, solvents, other drug use (such as non-prescription drugs including hallucinogens, stimulants, methylphenidate and designer drugs) and unspecified drug use. Recorded substance use included those used prior to and following pregnancy recognition.

Maternal cigarette smoking status was recorded during antenatal visits through self-report. If smoking was reported throughout or during part of the current pregnancy, the mother was considered a ‘current smoker’.

The following covariates were examined in this study: the maternal variables of prior neonatal death, stillbirths, prior low birthweight newborn and/or major congenital anomalies; maternal history of any mental illness; second-hand smoke exposure; and the neonatal variables of administration of oxygen, intermittent positive pressure ventilation (IPPV) mask and chest compression for resuscitation.

The following adverse neonatal outcomes and pregnancy complications were examined: “Fetal Alcohol Syndrome” (FAS) (ICD-10: Q86.0); “Fetus and newborn affected by maternal use of alcohol” (ICD-10: P04.3); 38 additional adverse neonatal outcomes identified through literature review on prenatal alcohol and other substance-exposed pregnancies (Supplementary File 1); pregnancy complications of bleeding < 20 weeks; antepartum hemorrhaging (bleeding ≥20 weeks); and intrauterine growth restriction (IUGR) (binary variable).

Maternal age during pregnancy was calculated by subtracting the maternal year of birth from the beginning of the fiscal year in which the birth occurred. Please see Supplementary File 2 for further information on data linkage and data cleaning performed in this study.

### Statistical analysis

Yearly prevalence of pregnancies in which maternal alcohol use was identified as a risk factor was calculated as a sum of the number of alcohol-exposed births as indicated by alcohol use identified as a risk, divided by the total number of live births in each fiscal year. The period prevalence of pregnancies in which maternal alcohol use was identified as a risk factor was calculated for the entire study population by dividing the number of alcohol-exposed births by the total number of births during the study period (Jan 1, 2015 – Mar 31, 2018).

Statistical analyses were conducted using STATA 16 and R. Descriptive statistics were generated for demographic variables. Chi-square tests were used for testing relationships between categorical variables: alcohol-exposed and non-exposed pregnancies for pregnancy complications and adverse neonatal outcomes. Crude odds ratios were generated for pregnancy complications and adverse neonatal outcomes. Odds ratios (OR) were generated using Chi-square tests and conditional maximum likelihood estimation (Fisher) was used for comparisons where expected cell counts were below 5.

Logistic regression (LR) was used to identify risk factors for alcohol use during pregnancy. Variables in the data such as maternal demographic information, parity, number of living children, prior neonatal death, prior stillbirth, prior low birthweight newborn, prior congenital anomaly, history of mental illness and number of antenatal visits were included in the models. Previous literature has shown that these variables may be associated with adverse maternal and neonatal conditions related to PAE. As such, these variables, as well as maternal smoking status and any substance use, were treated as confounders and were adjusted for in LR models to produce adjusted odds ratios for associations between alcohol use identified as a risk during pregnancy and i.) pregnancy complications; and ii.) adverse neonatal outcomes. Variables below the threshold of α = 0.05 were included in this regression model.

Lastly, logistic regression was used to examine whether there was an association between binge drinking and the three neonatal outcomes with the greatest frequency, controlling for the variables included in the previous regression model.

## Results

The final study population included 142,545 maternal records linked to 144,785 neonatal records that were discharged between January 1, 2015 and March 31, 2018 [17]. There were 6 observations that were excluded due to sex of the newborn being undifferentiated (*n* = 2), parity being unknown (*n* = 3) and maternal record with no corresponding neonatal record (*n* = 1), resulting in a final study population of 144,779 neonatal records. The average maternal length of stay in hospital was 61.37 h (2.5 days), there were 65,497 women (45.9%) who were primiparous, and the mean number of antenatal visits was 9.44 (Table 1).

**Table 1:** Characteristics of mothers by alcohol use during pregnancy identified as a risk factor

Variables	Alcohol use	No alcohol use	Overall	***P***-value
**N (%)**	1569 (1.1)	140,976 (98.9)	142,545	
Maternal age - mean (SD)	28.48 (5.7)	31.38 (5.2)	31.4 (5.2)	< 0.001^**^
Maternal total length of stay (hours) - mean (SD)	76.8 (156.9)	61.19 (77.6)	61.4 (79.0)	< 0.001^**^
Number of living children -mean (SD)	0.83 (1.21)	0.80 (1.01)	0.80 (1.01)	0.28
**Pregnancy history – n (%)**				
Parity = primiparous	821 (52.3)	64,676 (45.9)	65,497 (45.9)	< 0.001^**^
Prior neonatal death	10 (0.6)	413 (0.3)	423 (0.3)	0.024^*^
Prior stillbirth	14 (0.9)	998 (0.7)	1012 (0.7)	0.475
Prior low birthweight newborn (< 2500 g)	18 (1.1)	2116 (1.5)	2134 (1.5)	0.297
Major congenital anomalies (past pregnancy)	12 (0.8)	892 (0.6)	904 (0.6)	0.62
History of any mental illness	699 (44.6)	30,169 (21.4)	30,868 (21.7)	< 0.001^**^
**Current pregnancy**				
Number of antenatal visits - mean (SD)	9.02 (4.1)	9.44 (3.3)	9.44 (3.3)	< 0.001^**^
Total antenatal hospital admission (prior to delivery) - mean (SD)	0.15 (0.6)	0.09 (0.4)	0.09 (0.4)	< 0.001^**^
Bleeding (< 20 weeks) = Yes (%)	31 (2.0)	2596 (1.8)	2627 (1.8)	0.765
Antepartum hemorrhage > 20 weeks = Yes (%)	11 (0.7)	1804 (1.3)	1815 (1.3)	0.055
Intrauterine growth restriction (IUGR) = Yes (%)	40 (2.5)	3040 (2.2)	3080 (2.2)	0.328
**Substance use – n (%)**				
Any substance use	533 (34.0)	6268 (4.4)	6801 (4.8)	< 0.001^**^
Marijuana	388 (24.7)	4545 (3.2)	4933 (3.5)	< 0.001^**^
Cocaine	192 (12.2)	749 (0.5)	941 (0.7)	< 0.001^**^
Other drug(s)	104 (6.6)	629 (0.4)	733 (0.5)	< 0.001^**^
Prescription drugs	26 (1.7)	681 (0.5)	707 (0.5)	< 0.001^**^
Methadone	24 (1.5)	637 (0.5)	661 (0.5)	< 0.001^**^
Heroin	44 (2.8)	594 (0.4)	638 (0.4)	< 0.001^**^
Unspecified drug use	5 (0.3)	71 (0.1)	76 (0.1)	< 0.001^**^
Solvents	0 (0.0)	13 (0.0)	13 (0.0)	1
**Alcohol use**				
Alcohol during pregnancy identified as a risk = Yes (%)	1569 (100.0)	0 (0.0)	1569 (1.1)	< 0.001^**^
Number of drinks per week^a^ - mean (SD)	3.4 (9.6)	N/A	3.4 (9.6)	
Binge drinking^a^ – n (%)				< 0.001^**^
*Yes*	369 (23.5)	0 (0.0)	N/A	
*No*	751 (47.9)	0 (0.0)	N/A	
*Unknown*	449 (28.6)	0 (0.0)	N/A	
*N/A*	0 (0.0)	140,976 (100.0)	140,976 (100.0)	
**Cigarettes**				
Smoking during pregnancy				< 0.001^**^
Current	466 (29.7)	8094 (5.7)	8560 (6.0)	
Former	353 (22.5)	11,120 (7.9)	11,473 (8.0)	
Never (including unknown)	750 (47.8)	121,762 (86.4)	122,512 (85.9)	
Current number of cigarettes per day - mean (SD)	7.20 (47.8)	6.71 (5.1)	6.73 (5.2)	0.066
Exposure to second-hand smoke = Yes (%)	7.20 (6.0)	9466 (6.7)	9838 (6.9)	< 0.001^**^

Other drugs: Other non-prescription drugs including hallucinogens (lysergic acid diethylamide-LSD, magic mushrooms), stimulants (amphetamines, ephedrine, methamphetamine-ice, crystal-meth, methyldioxyamphetamine (MDA)), methylphenidate (Ritalin), designer drugs (Ketamine-dissociative anesthetic, ecstasy-serotonergic effects, Gamma-Hydroxybutyrate (GHB))

*N/A* Not applicable

^a^Among those with alcohol use identified as a risk factor

^*^Difference between alcohol exposed and non-alcohol exposed *p* < 0.05

^**^Difference between alcohol exposed and non-alcohol exposed *p* < 0.001

### Prevalence of pregnancies in which maternal alcohol use was identified as a risk factor

The prevalence of pregnancies wherein maternal alcohol use was identified as a risk factor by fiscal year in BC was 1.1, 1.1, 1.3 and 0.9% from the 2014/2015 to 2017/2018 fiscal years, respectively. There were 1593 alcohol-exposed neonates during the study period and 144,779 birth records; the resulting period prevalence of alcohol-exposed pregnancies in BC was 1.1 cases per 100 live births between January 1, 2015 and March 31, 2018.

### Characteristics of mothers who consumed alcohol during pregnancy versus mothers who did not consume alcohol during pregnancy

Mothers with alcohol use identified as a risk factor during pregnancy were significantly younger, had a longer length of stay in hospital, had a lesser number of antenatal visits and had a greater number of antenatal hospital admissions prior to delivery, had higher rate of a prior neonatal death and a history of any mental illness compared to mothers with no alcohol use identified as a risk factor during pregnancy (Table 1). In addition, mothers with alcohol use identified as a risk factor during pregnancy had a statistically significantly greater proportion of any substance use, current, former and second hand smoking compared to mothers who reported no alcohol use during pregnancy.

Table 2 presents logistic regression output, which demonstrated that being primiparous compared to multiparous, a greater number of living children, and maternal history of any mental illness were all statistically significantly associated with greater odds of maternal alcohol use during pregnancy. Older maternal age and a higher number of antenatal visits were statistically significantly associated with lesser odds of alcohol use identified as a risk factor during pregnancy.

**Table 2:** Logistic regression between maternal attributes and alcohol use during pregnancy identified as a risk factor

Risk factor	OR	Lower 95% CI	Upper 95% CI	***P***-value
Maternal Age	0.91	0.90	0.92	< 0.001^**^
Parity primiparous (relative to multiple)	1.51	1.30	1.76	< 0.001^**^
Number of living children^a^	1.26	1.18	1.34	< 0.001^**^
Prior neonatal death	1.86	0.78	3.73	0.116
Prior stillbirth	1.02	0.48	1.87	0.952
Prior low birthweight newborn (< 2500 g)	0.65	0.37	1.05	0.104
Prior major congenital anomaly	1.1	0.52	2.04	0.786
Maternal history of any mental illness	2.62	2.35	2.92	< 0.001^**^
Number of antenatal visits^a^	0.96	0.95	0.98	< 0.001^**^

^a^This was run as a continuous variable; the OR represents the effect size for each unit increase, or the odds for alcohol use being identified as a risk factor in each maternal record

^**^Significance of produced odds ratio has alpha = *p* < 0.001

### Adverse neonatal outcomes

Table 3 presents characteristics of newborns for the entire study population, stratified by alcohol use being identified as a risk factor by the antenatal care provider. On average, neonates with identified prenatal alcohol exposure were statistically significantly shorter at birth, had smaller head circumference, were less likely to be breastfed within the first hour of life and had higher rates of interventions: resuscitation/stabilization, oxygen for resuscitation, IPPV mask for resuscitation, than those who were alcohol non-exposed.

**Table 3:** Characteristics of newborns by alcohol use during pregnancy identified as a risk factor

Variables	Alcohol-exposed	Non alcohol-exposed	Overall	***P***-value
N (%)	1593 (1.1)	143,186 (98.9)	144,779	
Gestational age (weeks) - mean (SD)	38.3 (2.2)	38.3 (2.2)	38.4 (2.0)	0.077
Sex (%)				0.616
Female	795 (49.9)	69,753 (48.7)	70,548 (48.7)	
Male	798 (50.1)	73,426 (51.3)	74,224 (51.3)	
Other	0 (0.0)	7 (0.0)	7 (0.0)	
Birth length (cm) - mean (SD)	50.7 (3.4)	50.9 (3.0)	50.9 (3.0)	0.003^*^
Birth head circumference (cm) - mean (SD)	34.5 (1.9)	34.7 (1.8)	34.7 (1.8)	< 0.001^**^
IUGR – n (%)	42 (2.6)	3418 (2.4)	3460 (2.4)	0.51
NICU days (level II) - mean (SD)	7.80 (9.3)	8.41 (12.0)	8.40 (11.9)	0.541
NICU days (level III) - mean (SD)	8.79 (12.2)	15.98 (27.1)	15.89 (27.0)	0.195
Transfer NICU days (level II) - mean (SD)	3.52 (9.2)	3.10 (8.5)	3.10 (8.5)	0.604
Transfer NICU days (level III) - mean (SD)	3.37 (16.2)	1.60 (9.3)	1.62 (9.4)	0.051
Breastfeeding initiation - n (%)				< 0.001^**^
< = 1 h	815 (51.2)	83,205 (58.1)	84,020 (58.0)	
> 1 and < = 24 h	499 (31.3)	42,984 (31.3)	43,483 (30.0)	
> 24 h	49 (3.1)	3427 (2.4)	3476 (2.4)	
N/A	156 (9.8)	4756 (3.3)	4912 (3.4)	
Unknown	74 (4.6)	8814 (6.2)	8888 (6.1)	
Newborn resuscitation/ re-stabilization				
Drugs – n (%)				0.029^*^
Yes	19 (1.2)	932 (0.7)	951 (0.7)	
No	1573 (98.7)	142,149 (99.3)	143,722 (99.3)	
Unknown	1 (0.1)	105 (0.1)	106 (0.1)	
Oxygen - n (%)	153 (9.6)	8842 (6.2)	8995 (6.2)	< 0.001^**^
IPPV mask given - n (%)	170 (10.7)	9441 (6.6)	9611 (6.6)	< 0.001^**^
Chest compressions given - n (%)	4 (0.3)	173 (0.1)	177 (0.1)	0.263

Results are not adjusted for any confounders

*N/A* Not applicable - newborn was not breastfed or died in first two days of life, *NICU* Neonatal Intensive Care Unit, *IUGR* Intrauterine growth restriction, *IPPV* intermittent positive pressure ventilation

^*^Difference between alcohol exposed and non-alcohol exposed *p* < 0.05

^**^Difference between alcohol exposed and non-alcohol exposed *p* < 0.001

Table 4 presents overall counts of diagnoses received by newborns and counts stratified by maternal alcohol use identified as a risk factor during pregnancy. The 5 most prevalent ICD-10 diagnoses among all newborns were P07.1: “Other low birth weight (1000-2499g)” (5.5%), P28.5: “Respiratory failure of newborn” (4.3%), P05.9: “Slow fetal growth, unspecified” (2.7%), P92.8: “Other feeding problems of newborn” (1.6%) and P22.9: “Respiratory distress of newborn, unspecified” (1.4%).

**Table 4:** Counts of neonatal diagnoses by ICD-10 code among newborns by alcohol use during pregnancy identified as a risk factor

ICD-10 Code	Description	Alcohol-exposed n (%)	Non alcohol-exposed n (%)	Overall n (%)	Difference in proportion	***P***-value
Q86.0	Fetal alcohol syndrome (dysmorphic)	2 (0.1)	3 (0.0)	5 (< 0.1)	0.001	< 0.001^**^
P04.3	Fetus and newborn affected by maternal use of alcohol	18 (1.1)	12 (0.0)	30 (< 0.1)	0.011	< 0.001^**^
P05.9	Slow fetal growth, unspecified^a^	60 (3.8)	3906 (2.7)	3966 (2.7)	0.011	0.014^*^
P07.1	Other low birth weight (1000-2499 g)	137 (8.6)	7769 (5.4)	7906 (5.5)	0.032	< 0.001^**^
P22.0	Respiratory distress syndrome	24 (1.5)	1672 (1.2)	1696 (1.2)	0.003	0.257
P22.8	Other respiratory distress of newborn	18 (1.1)	598 (0.4)	616 (0.4)	0.007	< 0.001^**^
P22.9	Respiratory distress of newborn, unspecified	28 (1.8)	2063 (1.4)	2091 (1.4)	0.004	0.343
P24.0	Neonatal aspiration of meconium	4 (0.3)	474 (0.3)	478 (0.3)	0.000	0.739
P28.5	Respiratory failure of newborn	72 (4.5)	6150 (4.3)	6222 (4.3)	0.002	0.706
P28.9	Respiratory condition of newborn, unspecified	3 (0.2)	139 (0.1)	142 (0.1)	0.001	0.45
P29.9	Cardiovascular disorder originating in perinatal period, unspecified	0 (0.0)	22 (0.0)	22 (< 0.1)	0.000	1
P36	Bacterial sepsis of newborn	9 (0.6)	386 (0.3)	395 (0.3)	0.003	0.045^*^
P90	Convulsions of newborn	4 (0.3)	134 (0.1)	138 (0.1)	0.002	0.106
P92.5	Neonatal difficulty in feeding at breast	27 (1.7)	941 (0.7)	968 (0.7)	0.010	< 0.001^**^
P92.8	Other feeding problems of newborn	22 (1.4)	2255 (1.6)	2277 (1.6)	−0.002	0.605
P92.9	Feeding problems, unspecified	25 (1.6)	898 (0.6)	923 (0.6)	0.010	< 0.001^**^
P94.1	Congenital hypertonia	0 (0.0)	18 (0.0)	18 (0.1)	0.000	1
P96.1	Neonatal withdrawal symptoms from maternal use of drugs of addiction	27 (1.7)	630 (0.4)	657 (0.5)	0.013	< 0.001^**^
P96.8	Other specified conditions originating in the perinatal period	6 (0.4)	176 (0.1)	182 (0.1)	0.003	0.013^*^
Q05.9	Spina bifida, unspecified	0 (0.0)	8 (0.0)	8 (< 0.1)	0.000	1
Q24.9	Congenital malformations of heart, unspecified; includes anomaly or disease	1 (0.1)	41 (0.0)	42 (< 0.1)	0.001	0.955
Q35.9	Cleft palate, unspecified	1 (0.1)	57 (0.0)	58 (< 0.1)	0.001	1
Q36	Cleft lip without cleft palate	1 (0.1)	36 (0.0)	37 (< 0.1)	0.001	0.884
Q37	Cleft lip with cleft palate	1 (0.1)	74 (0.1)	75 (0.1)	0.000	1
Q89.7	Multiple congenital malformations, not elsewhere classified	2 (0.1)	10 (0.0)	12 (< 0.1)	0.001	< 0.001^**^
Q89.9	Congenital malformation, unspecified	0 (0.0)	7 (0.0)	7 (< 0.1)	0.000	1
R25.1	Tremor, unspecified	1 (0.1)	34 (0.0)	35 (< 0.1)	0.001	0.852
R62.8	Other lack of expected normal physiological development; includes: failure to gain weight, failure to thrive, infantilism NOS, lack of growth & physical retardation	0 (0.0)	29 (0.0)	29 (< 0.1)	0.000	1
R68.1	Nonspecific symptoms peculiar to infancy; includes: excessive crying of infant & irritable infant	0 (0.0)	109 (0.1)	109 (0.1)	−0.001	0.521

The following ICD-10 Codes were identified but there were no diagnoses among the study population: G47.0 Disorders of initiating and maintaining sleep [insomnias], J30.0 Vasomotor rhinitis, K59.1 Functional Diarrhoea, P04.4 Newborn affected by maternal use of other drugs of addiction, P05.1 Small for gestational age (<10th percentile), R06.7 Sneezing, R78.0 Excess alcohol blood level, R95 Sudden infant death syndrome, Z37.1 Stillbirth

^a^includes: fetal growth restriction NOS, Intrauterine fetal growth restriction, light for dates, light for gestational age (usually referred to weight below but length above 10th percentile), small and light for dates, small for dates, small for gestational (usually referred to weight and length below 10th percentile)

^*^Difference between alcohol exposed and non-alcohol exposed *p* < 0.05

^**^Difference between alcohol exposed and non-alcohol exposed *p* < 0.001

There were 5 neonatal records (0.003%) with a diagnosis of “Fetal alcohol syndrome” (FAS; ICD-10: Q86.0) in total among children born in this time period. Interestingly, in 3 of the 5 corresponding pregnancies, alcohol use was not identified as a risk by the antenatal care provider. Additionally, there were 30 neonatal records (0.02%) with a diagnosis of “Fetus and newborn affected by maternal use of alcohol” (ICD-10: P04.3), and 12 out of 30 were not identified as alcohol-exposed based on the same criteria.

Compared to pregnancies without reported alcohol use, pregnancies with alcohol use identified as a risk factor were statistically significantly associated with a greater proportion of the following ICD-10 diagnoses: P04.3: “Fetus and newborn affected by maternal use of alcohol”; P05.9: “Slow fetal growth, unspecified”; P07.1: “Other low birth weight (1000-2499g)”; P22.8: “Other respiration distress of newborn”; P36: “Bacterial sepsis of newborn”; P92.5: “Neonatal difficulty in feeding at breast”; P92.9: “Feeding problems, unspecified”; P96.1: “Neonatal withdrawal symptoms from maternal use of drugs of addiction”; P96.8: “Other specified conditions originating in the perinatal period”; Q86.0: “Fetal alcohol syndrome (dysmorphic)”; and Q89.7: “Multiple congenital malformations, not elsewhere classified” (Table 4).

Table 5 presents the crude and adjusted odds ratios between prenatal alcohol exposure identified as a risk factor and each adverse neonatal outcome, adjusted for maternal age, maternal smoking status, any maternal substance use, parity, prior neonatal deaths, prior stillbirth and prior low birthweight newborn, maternal history of any mental illness and the number of antenatal visits.

**Table 5:** Adjusted Odds Ratio of adverse neonatal outcomes associated with alcohol use during pregnancy identified as a risk factor

ICD-10 Code	Description	Odds Ratio	Adjusted Odds Ratio^**a**^	Lower 95% CI	Upper 95% CI	***p***-value
P04.3	Fetus and newborn affected by maternal use of alcohol	135.84	55.93	21.11	158.32	< 0.001^**^
P05.9	Slow fetal growth, unspecified^b^	1.40	1.07	0.79	1.41	0.648
P07.1	Other low birth weight (1000-2499 g)	1.64	1.25	1.01	1.53	0.033^*^
P22.0	Respiratory distress syndrome	1.29	1.01	0.62	1.56	0.95
P24.0	Neonatal aspiration of meconium	0.76	0.72	0.22	1.71	0.52
P22.8	Other respiratory distress of newborn	2.72	2.57	1.52	4.07	< 0.001^**^
P22.9	Respiratory distress of newborn, unspecified	1.22	0.98	0.64	1.42	0.90
P28.5	Respiratory failure of newborn	1.05	0.90	0.68	1.16	0.419
P28.9	Respiratory condition of newborn, unspecified	1.94	0.56	0.03	2.61	0.577
P29.9	Cardiovascular disorder originating in perinatal period, unspecified	0.00	0.00	N/A	N/A	0.97
P36	Bacterial sepsis of newborn	2.10	1.66	0.74	3.21	0.17
P90	Convulsions of newborn	2.69	1.58	0.47	3.92	0.385
P92.5	Neonatal difficulty in feed at breast	2.61	1.97	1.27	2.92	0.001^*^
P92.8	Other feeding problems of newborn	0.88	0.67	0.40	1.05	0.10
P92.9	Feeding problems, unspecified	2.53	2.06	1.31	3.09	< 0.001^**^
P94.1	Congenital hypertonia	0.00	0.00	N/A	N/A	0.99
P96.1	Neonatal withdrawal symptoms from maternal use of drugs of addiction	3.90	0.37	0.21	0.60	< 0.001^**^
P96.8	Other specified conditions originating in the perinatal period	3.07	2.02	0.70	4.58	0.13
Q05.9	Spina bifida, unspecified	0.00	0.00	N/A	N/A	1.00
Q24.9	Congenital malformations of heart, unspecified; includes anomaly or disease	2.19	5.34	0.30	25.58	0.100
Q35.9	Cleft palate, unspecified	1.58	1.42	0.08	7.08	0.74
Q36	Cleft lip without cleft palate	2.50	2.30	0.13	11.69	0.43
Q37	Cleft lip with cleft palate	1.21	1.02	0.06	4.87	0.98
Q86.0	Fetal alcohol syndrome (dysmorphic)	59.97	10.62	0.36	13.05	0.100
Q89.7	Multiple congenital malformations, not elsewhere classified	18.00	15.63	1.99	79.37	0.003^*^
Q89.9	Congenital malformation, unspecified	0.00	0.00	N/A	N/A	0.990
R25.1	Tremor, unspecified	2.64	1.46	0.08	7.38	0.718
R62.8	Other lack of expected normal physiological development; includes: failure to gain weight, failure to thrive, infantilism NOS, lack of growth & physical retardation	0.00	0.00	N/A	N/A	0.990
R68.1	Nonspecific symptoms peculiar to infancy; includes: excessive crying of infant & irritable infant	0.00	0.00	N/A	6.78	0.975

^a^adjusted for maternal age, maternal smoking status, any maternal substance use, parity, prior neonatal deaths, prior stillbirth and prior low birthweight newborn, maternal history of any mental illness and the number of antenatal visits

^b^includes: fetal growth restriction NOS, Intrauterine fetal growth restriction, light for dates, light for gestational age (usually referred to weight below but length above 10th percentile), small and light for dates, small for dates, small for gestational (usually referred to weight and length below 10th percentile)

^*^Significance of produced odds ratio has alpha = *p* < 0.05

^**^Significance of produced odds ratio has alpha = *p* < 0.001

**Fig. 1:**
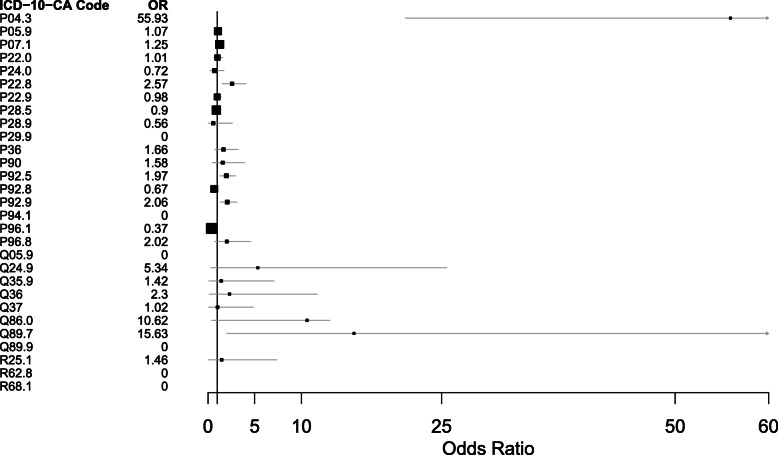
Forest Plot of adjusted Odds Ratios for the association between selected adverse neonatal outcomes and alcohol use during pregnancy identified as a risk factor. P04.3 - Fetus and newborn affected by maternal use of alcohol; P05.9 - Slow fetal growth, unspecified; P07.1 - Other low birth weight (1000-2499 g); P22.0 - Respiratory distress syndrome; P22.8 - Other respiratory distress of newborns; P22.9 - Respiratory distress of newborn, unspecified; P24.0 - Neonatal aspiration of meconium; P28.5 - Respiratory failure of newborn; P28.9 - Respiratory condition of newborn, unspecified; P29.9 - Cardiovascular disorder originating in perinatal period, unspecified; P36 - Bacterial sepsis of newborn; P90 - Convulsion of Newborn; P92.5 - Neonatal difficulty in feed at breast; P92.8 - Other feeding problems of newborn; P92.9 - Feeding problems, unspecified; P94.1 - Congenital Hypertonia; P96.1 - Neonatal withdrawal symptoms from maternal use of drugs of addiction; P96.8 - Other specified conditions originating in the perinatal period; Q05.9 - Spina bifida, unspecified; Q24.9 - Congenital malformations of heart, unspecified; includes anomaly or disease; Q35.9 - Cleft palate, unspecified; Q36 - Cleft lip without cleft palate; Q37 - Cleft lip with cleft palate; Q89.7 - Multiple congenital malformations, not elsewhere classified; Q89.9 - Congenital Malformation, unspecified; R25.1 - Tremor, unspecified; R62.8 - Other lack of expected normal physiological development; includes: failure to gain weight, failure to thrive, infantilism NOS, lack of growth & physical retardation; R68.1 - Nonspecific symptoms peculiar to infancy; includes: excessive crying of infant & irritable infant

After adjustment, neonates with prenatal alcohol exposure identified as a risk factor had 1.25 times greater odds (aOR = 1.25; 95% CI: 1.01, 1.53; *p* = 0.033) of being diagnosed with P07.1: “Other low birth weight (1000-2499g)”, 2.57 times greater odds (aOR = 2.57; 95% CI: 1.52, 4.07; *p* < 0.001) of being diagnosed with P22.8: “Other respiration distress of newborn”, 1.97 times greater odds (aOR = 1.97; 95% CI: 1.27, 2.92; *p* < 0.001) of being diagnosed with P92.5: “Neonatal difficulty feeding at breast”, 2.06 times greater odds (aOR = 2.06; 95% CI: 1.31, 2.92; *p* < 0.001) of being diagnosed with P92.9: “Feeding problems, unspecified” and 15.63 times greater odds (aOR = 15.63; 95% CI: 1.99, 79.37; *p* < 0.003) of being diagnosed with Q89.7: “Multiple congenital malformations, not elsewhere classified” compared to neonates without reported prenatal alcohol exposure (Table 5 and Fig. 1).

Table 6 presents the adjusted odds ratios for the effects of binge drinking on adverse neonatal outcomes. The three most frequent adverse neonatal outcomes included: P05.9: “Slow fetal growth, unspecified”, P07.1: “Other low birth weight (1000-2499g)” and P28.5: “Respiratory failure of newborn”. Of the three outcomes evaluated, the only statistically significant association was between binge drinking and P28.5: “Respiratory failure of newborn”. Neonates whose mother reported binge drinking during pregnancy had 2.03 times greater odds (aOR = 2.03; 95% CI: 1.02, 4.10; *p* = 0.044) of being diagnosed with P28.5: “Respiratory failure of newborn” compared to those neonates whose mothers did not report binge drinking (Table 6).

**Table 6:** Adjusted Odds Ratios of adverse neonatal outcomes associated with binge drinking among pregnancies where alcohol use was identified as a risk factor (*n* = 1569)

Outcome	Exposure	Adjusted Odds Ratio^**a**^	Lower 95% CI	Upper 95% CI	***P***-value
P05.9: Slow fetal growth, unspecified	Binge drinking = No (reference group)	–	–	–	–
	Binge Drinking = Yes	2.00	0.95	4.31	0.071
	Binge Drinking = Unknown	2.00	0.92	4.20	0.081
P07.1: Other low birth weight (1000-2499 g)	Binge drinking = No (reference group)	–	–	–	–
	Binge Drinking = Yes	1.59	0.94	2.68	0.083
	Binge Drinking = Unknown	1.53	0.94	2.52	0.089
P28.5: Respiratory failure of newborn	Binge drinking = No (reference group)	–	–	–	–
	Binge Drinking = Yes	2.03	1.02	4.10	0.044
	Binge Drinking = Unknown	1.88	0.95	3.77	0.071

^a^adjusted for maternal age, maternal smoking status, any maternal substance use, parity, prior neonatal deaths, prior stillbirth and prior low birthweight newborn, maternal history of any mental illness and the number of antenatal visits

### Pregnancy complications

During the study period, there were 2712 (1.9%) pregnancies where bleeding < 20 weeks was reported, 1862 (1.3%) pregnancies where antepartum hemorrhage > 20 weeks was reported and 3460 (2.4%) pregnancies where IUGR was identified as a risk. There were no statistically significant differences in the prevalence or odds of pregnancy complications between prenatally alcohol-exposed and non-exposed pregnancies (Table 7).

**Table 7:** Odds Ratios of pregnancy complications associated with alcohol use identified as a risk factor during pregnancy

Pregnancy complication	OR	Lower 95% CI	Upper 95% CI	***p***-value
Bleeding (< 20 weeks)	1.14	0.79	1.61	0.40
Antepartum hemorrhage > 20 weeks	0.58	0.30	1.02	0.06
Intrauterine growth restriction (IUGR)	1.11	0.79	1.51	0.51

## Discussion

### Main findings

The prevalence of alcohol use identified as a risk factor ranged between 0.9 and 1.3% among BC pregnancies in the years examined. While this is comparable to a prevalence estimate of alcohol use (any amount) of 1.8% from an Ontario birth registry [18], it is notably lower as compared to a regional estimate of alcohol use (any amount) of 10% in 2015 in Manitoba [19]; of which, the latter was based on post-delivery discharge follow-up by public health nurses [19]. This illustrates that provincial birth registries on their own, including PSBC, may be underestimating alcohol use behaviour during pregnancy, and post-partum measures may be able to collect more accurate information. The estimates based solely on birth registry information in BC and ON are remarkably lower compared to the 10% national prevalence estimate of alcohol use during pregnancy across Canada based on a meta-analysis of existing epidemiological studies, which included studies using self-report in survey responses [20]. There is heterogeneity between provinces in how data on alcohol use during pregnancy are collected and recorded, including the alcohol use definition and timing of data collection. Though birth registry data in BC and Ontario generate similar estimates, these data are not comparable: the BCPDR has operationalized alcohol use differently by emphasizing that alcohol use is not only self-reported, but also must be deemed a risk factor in the respective pregnancy [21] and is solely based on that which occurred following pregnancy recognition, captured in a binary data element.

This population-based study found specific maternal risk factors for alcohol use identified as a risk factor during pregnancy that may be useful for targeting education and prevention efforts such as mothers’ age, parity, prior neonatal death, and history of any mental illness. In addition, there was a greater prevalence of other substance use, current and former smoking status, and exposure to second-hand smoke among women with alcohol use identified as a risk factor.

Results also highlighted factors that might identify newborns with prenatal alcohol exposure who are at risk for low birth weight, respiratory distress, and feeding difficulties. Our results did not find any differences between neonates with and without identified prenatal alcohol exposure for the three pregnancy complications examined: bleeding before 20 weeks, antepartum hemorrhaging (bleeding ≥20 weeks) and IUGR. During the neonatal period, however, low birth weight (adjusted), shorter length, and smaller head circumference (unadjusted) were found to be more common for the alcohol-exposed group.

In this study, women with mean ages between 28 and 31 years were at higher risk for alcohol use identified as a risk factor during pregnancy. They were also more likely to be giving birth to their first child and to attend fewer antenatal care visits. Together, these factors indicate that women at risk for consuming alcohol in pregnancy may receive less healthcare support and information during pregnancy due to fewer medical encounters. This suggests that primary healthcare providers may wish to consider greater screening and possible referral to more extensive supports and education for women in this demographic. This is especially important if antenatal alcohol use behaviour in the first pregnancy is predictive of use behaviour while breastfeeding, or during subsequent pregnancies.

### Interpretation

During the timeframe of this study, we found a period prevalence of 1.1% for pregnancies where alcohol use was identified as a risk factor. Prevalence for the prior year (2014/2015) in the same jurisdiction was 1.2% [22] and it has ranged over time from 1.3% in 2000/2001 [23] to 0.4% in 2012/2013 [24]. Although some research has suggested a trend towards declining rates of maternal alcohol consumption during pregnancy in both Canada and the United States [25, 26], the data are variable and complex due to factors such as the population studied, data collection methodology, and alcohol use definitions in each study. Globally, however, there is reason to believe that rates of alcohol-exposed pregnancies may be increasing overall [5].

All women who are pregnant or considering pregnancy should be engaged by their primary healthcare providers in discussion regarding substance use, possible harms, and interventions [3]. It may be especially important, however, to provide this guidance and support to first-time mothers, women in particular age-groups, and those with previous mental health issues, based on the findings in the current study. Previous research has reported that women with mental health issues receive less prenatal care than those without mental health issues [27], suggesting a complex relationship between mental health problems, obtaining prenatal care, and alcohol consumption in pregnancy. Women with a history of mental health issues may have an elevated risk for substance use for a number of reasons, including a history of trauma, interpersonal issues, or adverse psycho-social factors [28]. Embedding prenatal education, care, and support into mental health settings may be one possible direction for strategies aimed at reducing PAE and increasing engagement with antenatal healthcare services.

Interestingly, several maternal factors associated with alcohol use identified as a risk factor during pregnancy in this study were similar to those previously reported for cannabis use in pregnancy. Luke et al. [29] noted that a history of mental health issues was more common in women who used cannabis in pregnancy. They also found that the cannabis use group were more likely to use alcohol and tobacco as well, suggesting that much like in the present study, infants exposed to one substance were also frequently exposed to other substances, likely increasing their risk for adverse outcomes. Healthcare providers should therefore be encouraged to consider screening for additional substances if alcohol use is reported or suspected during pregnancy.

These findings may be reflective of the mixed results for growth deficits associated with PAE found in other studies [3, 30] or it may suggest challenges with growth were either not present or undetected in the earlier periods of pregnancy. Coles [2] noted that while some growth deficit can occur in the first and second trimester of alcohol-exposed pregnancies, a more pronounced deficit in weight, length, and head circumference occurs in the third trimester. In the context of this study, it could be that earlier healthcare encounters were less able to identify IUGR and only noted growth deficits at or after birth. As well, given that smoking is well known to influence fetal growth [30, 31] and 29.7% of the women in the study who had alcohol use identified as a risk factor also had concurrent tobacco use, compared to only 5.7% among women for whom alcohol use was not identified as a risk factor; therefore, it is possible that patterns of smoking affected or confounded growth results. Regardless, outcomes from this study suggest that even when growth deficits are not identified early in the pregnancy, primary care providers may wish to consider increased monitoring of fetus development in mothers who are believed to be tobacco and/or alcohol users.

Infants exposed to alcohol identified as a risk factor in this study also showed greater early difficulties with feeding, need for resuscitation, more congenital abnormalities, and longer hospital stays suggesting some important vulnerabilities and risks. Also, these infants were more often administered oxygen or IPPV and more often had problems initiating breastfeeding or were not breastfed at all. Binge drinking during pregnancy was significantly associated with “Respiratory failure of newborn” (ICD-10: P28.5), but not with “Slow fetal growth, unspecified” (ICD-10: P05.9) or “Other low birth weight (1000-2499g)” (ICD-10: P07.1). These results, however, should be interpreted with caution because the measure on binge drinking has not been validated.

Only a small proportion of infants with identified prenatal alcohol exposure as a risk factor, however, received a diagnosis of FAS. As well, many of the mothers of children identified as affected by alcohol at birth did not report alcohol use to their antenatal healthcare providers; this may indicate that alcohol use occurred before pregnancy recognition, it was simply omitted, or was never screened for. This presents a potential challenge for healthcare providers in identifying and channeling infants at risk into early intervention programs for PAE. According to the new Canadian diagnostic guidelines (2016) for Fetal Alcohol Spectrum Disorder (FASD), infants may now be given a designation of “At Risk for FASD” if they have known PAE identified through the three sentinel facial features of FASD or reported by a reliable source, in addition to microcephaly at birth [3]. Having this designation available may provide an important opportunity for early detection of infants not receiving a full FAS diagnosis at birth. As the “At Risk for FASD” designation is a new diagnostic tool only added to the Guidelines recently, it will be of interest for future research studies to establish whether it is effective at directing these at-risk infants and their families into supports and early interventions.

### Strengths and limitations

This study has several important strengths. This is a recent, population-based study with a large, representative sample size, and is the first study to examine maternal and neonatal outcomes in relation to alcohol use identified as a risk factor among pregnancies in BC. This study has important findings, which must be understood within the context of several existing limitations. First, alcohol use identified as a risk factor during pregnancy was recorded during antenatal visits by the antenatal care provider, and there may be subjectivity in this identification. It may be the case that these data were ascertained in a biased manner on an individual and/or systemic basis. For example, some women who used alcohol during pregnancy may have not been screened, while other women with medical risk factors or in special sub-populations, for example, may have been screened more heavily for alcohol use. Second, due to the relatively small number of women with alcohol use identified as a risk factor during pregnancy, those with both low and high use were placed into a single group for analysis. As a result, possible dose-dependent outcomes could not be examined. As well, outcomes associated with binge drinking, which is known to produce more damaging results [1, 32], were not examined separately due to low numbers reported. The exact sensitivity of alcohol use screening as collected in the BCPDR is unknown, though it is plausible that higher levels of alcohol use following pregnancy recognition may be more likely to be recorded as being a risk. Binge drinking itself, however, was measured among women with alcohol use identified as a risk factor. The validity of the data on maternal alcohol use in the BCPDR is unknown, though it is not routinely used in surveillance, nor is it mandatory to enter into the information system [33]. Based on the sensitive nature, alcohol use identified as a risk factor during pregnancy may be comparable to maternal smoking status, which was found to have 63.9% sensitivity and 98.2% specificity in a validation study of the BCPDR [33]. Also, as noted above, multi-substance use was high and could have influenced outcomes in this study. In many cases, women who used alcohol in pregnancy also used tobacco, cannabis or other substances, making interpretation of the effect of alcohol alone more difficult. As alcohol consumption was self-reported by women, there is a possibility of social desirability bias influencing both reported usage and amounts consumed. Furthermore, the discrepancy between self-reported alcohol use during pregnancy and identification of alcohol use as a risk factor is unknown; however, all alcohol use during pregnancy is a risk factor for adverse neonatal outcomes. Since maternal self-report of alcohol use was solely based on that which occurred after pregnancy recognition, it is possible that PAE went unrecorded in many cases. This study was not able to examine the timing of pregnancy recognition, and therefore, the role of unplanned pregnancies is unknown. Lastly, FAS is rarely diagnosed at birth, therefore, data from birth registries will always underestimate FAS incidence.

## Conclusion

Understanding and describing how PAE is related to fetal growth continues to be challenging. Future research in this area may also wish to examine at what point in the pregnancy various types of growth deficits can be detected, levels of exposure necessary to produce specific deficits, and how multi-substance use contributes to this picture.

Birth registries across country should harmonize the definition and collection of alcohol use data and conduct post-discharge follow-up. Obstetricians and gynaecologists, as well as general practitioners and family physicians, are in a unique first point position, as they can screen childbearing age and pregnant women for alcohol use and identify pregnancies at risk for FASD. All prenatal care providers should consistently be trained to screen for alcohol use during pregnancy in accordance with the 2020 Society of Obstetricians and Gynaecologists of Canada (SOGC) guidelines [16]. Pregnant women with identified alcohol use should be provided with brief interventions, specialized treatment, support programs, and community resources [34]. All these strategies can significantly reduce prevalence of alcohol use during pregnancy and FASD.

## Supplementary Information

**Additional file 1.** Adverse neonatal outcomes identified through literature review on prenatal alcohol and other substance exposed pregnancies

**Additional file 2.** Supporting Information for Methodology

## Data Availability

The data that support the findings of this study are available from Perinatal Services BC, but restrictions apply to the availability of these data, which were used under license for the current study, and so are not publicly available. Data are however available from the authors upon reasonable request and with permission of Perinatal Services BC.

## Abbreviations

aOR: Adjusted odds ratio
BC: British Columbia
BCPDR: British Columbia Perinatal Data Registry
CI: Confidence interval
FAS: Fetal Alcohol Syndrome
FASD: Fetal alcohol spectrum disorder
ICD-10: The International Classification of Diseases, Version 10
IPPV: Intermittent positive pressure ventilation
IUGR: Intrauterine growth restriction
LR: Logistic regression
NICU: Neonatal intensive care unit
NOS: Not otherwise specified
OR: Odds ratio
PAE: Prenatal alcohol exposure
PSBC: Perinatal Services BC
SD: Standard deviation
SOCG: Society of Obstetricians and Gynaecologists of Canada
