# Supplementary material for: Maternal alcohol use, adverse neonatal outcomes and pregnancy complications in British Columbia, Canada: a population-based study
Source: BMC Pregnancy Childbirth. 2021 Jan 22;21:74. doi: 10.1186/s12884-021-03545-7 (PMC7821646; doi:10.1186/s12884-021-03545-7)
Supplement: Supplementary file 1 — Additional file 1. Adverse neonatal outcomes identified through literature review on prenatal alcohol and other substance exposed pregnancies [file 12884_2021_3545_MOESM1_ESM.docx]

**Maternal alcohol use, adverse neonatal outcomes and pregnancy complications in British Columbia, Canada: A population-based study**

**Additional File 1.** Adverse neonatal outcomes identified through literature review on prenatal alcohol and other substance exposed pregnancies

| **Code** | **ICD-10-CA 2012 description** |
| --- | --- |
| G47.0 | Disorders of initiating and maintaining sleep [insomnias] |
| J30.0 | Vasomotor rhinitis |
| K59.1 | Functional Diarrhoea |
| P04.3 | Fetus and newborn affected by maternal use of alcohol |
| P04.4 | Newborn affected by maternal use of other drugs of addiction |
| P05.1 | Small for gestational age (<10th percentile) |
| P05.9 | Slow fetal growth, unspecified; includes: fetal growth restriction NOS, Intrauterine fetal growth restriction, light for dates, light for gestational age (usually referred to weight below but length above 10th percentile), small and light for dates, small for dates, small for gestational (usually referred to weight and length below 10th percentile) |
| P07.1 | Other low birth weight (1000-2499g) |
| P22.0 | Respiratory distress syndrome |
| P22.8 | Other respiratory distress of newborns |
| P22.9 | Respiratory distress of newborn, unspecified |
| P24.0 | Neonatal aspiration of meconium |
| P28.5 | Respiratory failure of newborn |
| P28.9 | Respiratory condition of newborn, unspecified |
| P29.9 | Cardiovascular disorder originating in perinatal period, unspecified |
| P36 | Bacterial sepsis of newborn |
| P90 | Convulsion of Newborn |
| P92.5 | Neonatal difficulty in feed @ breast |
| P92.8 | Other feeding problems of newborn |
| P92.9 | Feeding problems, unspecified |
| P94.1 | Congenital Hypertonia |
| P96.1 | Neonatal withdrawal symptoms from maternal use of drugs of addiction |
| P96.8 | Other specified conditions originating in the perinatal period |
| Q05.9 | Spina bifida, unspecified |
| Q24.9 | Congenital malformations of heat, unspecified; includes anomaly or disease |
| Q35.9 | Cleft palate, unspecified |
| Q36 | Cleft lip without cleft palate |
| Q37 | Cleft lip with cleft palate |
| Q86.0 | Fetal alcohol syndrome (dysmorphic) |
| Q89.7 | Multiple congenital malformations, not elsewhere classified |
| Q89.9 | Congenital Malformation, unspecified |
| R06.7 | Sneezing |
| R25.1 | Tremor, unspecified |
| R62.8 | Other lack of expected normal physiological development; includes: failure to gain weight, failure to thrive, infantilism NOS, lack of growth & physical retardation |
| R68.1 | Nonspecific symptoms peculiar to infancy; includes: excessive crying of infant & irritable infant |
| R78.0 | Excess alcohol blood level |
| R95 | Sudden infant death syndrome |
| Z37.1 | Stillbirth |
