# Supplementary material for: Maternal alcohol use, adverse neonatal outcomes and pregnancy complications in British Columbia, Canada: a population-based study
Source: BMC Pregnancy Childbirth. 2021 Jan 22;21:74. doi: 10.1186/s12884-021-03545-7 (PMC7821646; doi:10.1186/s12884-021-03545-7)
Supplement: Supplementary file 2 — Additional file 2. Supporting Information for Methodology [file 12884_2021_3545_MOESM2_ESM.docx]

**Maternal alcohol use, adverse neonatal outcomes and pregnancy complications in British Columbia, Canada: A population-based study**

**Additional File 2.** Supporting Information for Methodology

*Maternal and neonatal records*

Data were merged for maternal and neonatal variables such that maternal and newborn records contained all respective discharge records and diagnoses. The data were then stacked using a linkage key to link neonatal records to respective mothers. For non-singleton pregnancies, a unique record was created for each non-singleton birth by linking individual neonatal records to respective mothers using the maternal linkage key. Data cleaning was done in accordance with PSBC standard protocol.

*Data cleaning steps*

Alcohol as a risk factor was coded as yes or no, such that no was an amalgamation of no, null, or not documented. Binge drinking was coded as yes, no, unknown or not available.

Substance use as a risk factor was coded as yes or no, such that no was an amalgamation of no, null, or not documented.

If the mother had reported smoking only prior to the current pregnancy and did not smoke during the current pregnancy, she was considered a ‘former smoker’. Otherwise, if the mother did not report smoking, she was considered a ‘never smoker’.

For the following covariates: prior neonatal death, stillbirths, previous low birthweight and/or major congenital anomalies; maternal history of any mental illness; second-hand smoke exposure; and administration of oxygen, intermittent positive pressure ventilation (IPPV) mask and chest compression for resuscitation. These covariates were coded as yes or no, such that no was an amalgamation of no, null or not documented. Drugs used for resuscitation or stabilization were both operationalized as “yes”, “no” or “unknown”.

For each adverse neonatal outcome (listed in Appendix S1), if the neonatal records contained the ICD-10 code of the adverse outcome, the coding was yes; otherwise it was no. A woman was considered as having IUGR if during the antenatal period the fetal weight, estimated by ultrasound, was <10th percentile and/or abdominal circumference was <10th percentile for gestational age.
